# How the brain structures the boundaries of psychiatric discourse

**DOI:** 10.3389/fpsyg.2026.1725149

**Published:** 2026-02-19

**Authors:** Christophe Gauld, Jean-Arthur Micoulaud-Franchi

**Affiliations:** 1 Department of Child Psychiatry, Hospices Civils de Lyon, Lyon, France; 2 SANPSY, CNRS, UMR 6033, Bordeaux, France; 3 Institut des Sciences Cognitives Marc Jeannerod, UMR 5229, CNRS, Université Claude Bernard Lyon 1, Lyon, France; 4 University Sleep Medicine Service, University Hospital of Bordeaux, Bordeaux, France

**Keywords:** boundary object, brain talk, critical psychiatry, metaphor, neuroscience

## Abstract

Psychiatry has long relied on the brain as both an explanatory model and a source of legitimacy, yet the persistence of this reference cannot be reduced to the progress of neuroscience. This Perspective explores how “brain talk” operates as a metaphorical and organizational device that structures the boundaries of psychiatric discourse. Drawing on the concepts of *boundary object* and *conceptual metaphor*, it argues that the brain has served less as a scientific endpoint than as an epistemic anchor enabling psychiatry to maintain coherence amid conceptual fragmentation and uncertainty. As a boundary object, the brain provides a shared referent through which heterogeneous communities (clinicians, researchers, and patients) can coordinate their practices despite divergent interests and languages. As a conceptual metaphor, it generates narratives that render psychiatric knowledge intelligible, productive, and culturally resonant, even when the underlying mechanisms remain opaque. Through this dual role, the cerebral metaphor contributes to psychiatry’s self-organization as a pluralistic field grounded in both scientific aspiration and epistemic humility. Thus, finally, this Perspective suggests that the power of brain discourse reveals less about the brain itself than about psychiatry’s ongoing effort to stabilize its identity and legitimacy through the metaphors.

## 1 Introduction

Since at least the seventeenth century (Suzuki, 1995), psychiatry has long oscillated between its scientific ambitions and its clinical and humanistic commitments, often grounding its discourse in references to the brain (Harrington, 2020). This cerebral anchoring, however, is not simply indicative of scientific progress; it also points to broader epistemological and sociological dynamics (Kendler et al., 2022; Kendler, 2025). In this Perspective, we explore how the metaphorical invocation of the brain in psychiatry can be understood not only as a narrative device, but also as a stabilizing force in a discipline marked by conceptual fragmentation and historical contingency. Based on the notions of boundary object and conceptual metaphor, we argue that brain talk plays a dual role: it coordinates diverse practices and produces narratable forms of knowledge, thus supporting psychiatry’s epistemic cohesion.

## 2 The brain as a boundary object

First, metaphorical brain talk has often functioned as a “promissory note” or a “working hypothesis” (Schneider, 1959; Kendler, 2025). Beyond this speculative role embedded in regimes of hope (Moreira and Palladino, 2005) and promises (Joly, 2010), it can also be understood as a potential centralizing epistemic object – what has been called a “boundary object” in sociology of science (Star and Griesemer, 1989) [or an “epistemic hub” in the context of psychiatric nosology (Keuck, 2011)]. A boundary object is defined as a concept that is adaptable to different perspectives while robust enough to maintain a shared identity across social worlds (Star and Griesemer, 1989). Considering the brain as a boundary object is one possible way to understand why clinical psychiatry has consistently turned to this organ. This repeated return may not necessarily be driven by the scientific findings of brain research, but rather by the fact that the brain has functioned as a stabilizing referent for heterogeneous communities invested in the same object of concern (aka, a subject, the psychiatric patient). Psychiatry’s relationship to the brain may thus appear less grounded in hopes for neuroscientific discoveries than as a historical and sociological regularity aimed at unifying the field’s heterogeneous communities. In other words, the brain may have operated as an obligatory passage point – a site where diverse actors, methods, and concepts converge and organize themselves, leading to a form of path dependency based on previously traveled routes (Callon, 1984). It centralizes not only the discourses of disciplines adjacent to psychiatry, but also provides a rationale for its legitimacy and credibility (Gieryn, 1983), while structuring scientific and clinical controversies (Raynaud, 2015; Gauld et al., 2022). This dynamic also reflects communication patterns in which the anticipated social influence of neuroscience extends beyond its demonstrated capacities, a process that reinforces the prominence of the brain even when empirical support remains uncertain for clinical practice (Schleim, 2014; Gough, 2023). In this way, the metaphor of the brain would function less through its explanatory power than through its epistemic utility as a central organizing hub; metaphorical brain talk may thus have enabled psychiatry to manage its internal diversity and external pressures. This use of the brain as a “coordinating referent” is compatible with a broadly nominalist view of psychiatric classification, in which diagnostic entities are primarily treated as epistemic tools that facilitate coordination, communication, and action across heterogeneous practices (Schleim, 2014). Importantly, this compatibility is invoked in a deliberately minimal sense and does not commit the present perspective to any specific version of nominalism, nor to a particular ontological or explanatory stance regarding the nature of psychiatric disorders. From this perspective, the brain supports classificatory work by virtue of its coordinating position, functioning as a hub between nosographic entities, and appears to have gained its prominence in the field precisely through this capacity to coordinate entities that are not necessarily natural kinds. Invoking the brain as a boundary object may reflect psychiatry’s epistemic vulnerability, in a strategic alignment with sciences of ignorance, where organizing around what is not known helps sustain disciplinary cohesion (Gross and McGoey, 2015). Moreover, this same boundary object [which does not function as a criterion for demarcating the boundaries of psychiatry (Brülde and Radovic, 2006; Gough, 2023)] could also apply to another specialty such as neurology, and it would then also help clarify its shared identities across its different social worlds.

## 3 The brain as a conceptual metaphor

In this way, secondly, developing narratives about our ignorance appears essential. Such narratives can be seen as meaning-laden discourses that contribute to the ongoing construction of psychiatry’s identity through metaphor. Conceptual metaphors can be defined by their meaning-making function, as they are acts of *poiesis* that constitute attempts to legitimate psychiatric disorders through culturally available figures (Kirmayer, 2023). While the boundary object helps to connect and unify fields in contexts of epistemic uncertainty, the metaphor enables the production of knowledge by drawing on narrative. For instance, the metaphors of the “chemical imbalance,” “network dysregulation,” “precision biomarkers” or “broken brain” are not (only) partially inaccurate explanations but productive fictions (Giere, 1988), allowing the brain to be narrated (as a boundary object) to support institutional alliances, research funding, clinical translation, or patient recognition. Their epistemic functions lies not in their truth-value (Moreira and Palladino, 2005), but in their capacity to align actors and resources by producing shareable forms of knowledge. To summarize, conceptual metaphors help stabilize heterogeneous and disparate meanings within a community by producing narrated forms of knowledge (Kirmayer, 2023). Of course, their use is not without risk, and from a critical and as has been widely discussed within the field of ignorance studies (Gross and McGoey, 2015), viewing psychiatric models as useful fictions may itself constitute an epistemic constraint, i.e., the redirection of attention and resources limiting the exploration of alternative questions. However, in many contemporary contexts, brain talk is no longer received or mobilized as metaphorical, but rather as a literal explanatory framework. Such a literalization is evident in the structure of psychiatric research and funding priorities, in public and media representations of psychiatric disorders, and in some subfields of clinical practices that implicitly treat psychiatric conditions as direct expressions of cerebral dysfunction.

## 4 Discussion

To conclude, as Rosenberg once observed, psychiatry might have suffered from an anxious sense of inferiority, lacking stable categories and clear “biopathological mechanisms” (Rosenberg, 2006). However, we argue here that metaphorical brain talk is not only a “transitional object,” a blanket, soft toy, or bit of cloth through which young psychiatry might have developed a secure attachment (Winnicott, 1953). Rather, and without seeking to pathologize the discipline, such discourse could be seen as the surface expression of a deeper dissociative dynamic. This dissociation is likely intrinsically constitutive and generative for psychiatry, in its proximity to imperfection, uncertainty, and humility in relation to what remains ungraspable. The two complementary tools described above (the boundary object and the conceptual metaphor) illustrate how the reassociation of psychiatry through brain discourse, within a framework of tolerant and dappled pluralism, may take shape at the level of scholarly communities, opening onto a logic of productive controversy within the discipline (Kendler, 2016, 2025). The present argument is not intended to advance a normative claim about the role the brain *should* play in psychiatry; rather, our position is descriptive and sociological: we aim to account for the fact that, whether one endorses it or not, brain talk has acquired a central position in psychiatry through historical path dependencies, institutional arrangements, and its social and community preeminence. The prominence of the brain, here, is therefore approached here as an empirical feature of the field’s organization.

To summarize, psychiatry is likely to require constant reconnection around a stabilizing epistemic referent, the boundary object, which must be narrated through metaphors. The rearticulation of psychiatry with the cerebral metaphor could function as an epistemic anchor, constituting an infrastructure that, despite its limitations, has historically contributed to stabilizing the multiple identities upon which psychiatry precariously depends.

Could one then imagine another psychiatric boundary object that, for instance, does not emerge from a biological discourse and is instead grounded in entities that are more directly and clinically tractable for the clinician? This suggestion is meant in a deliberately exploratory and non-prescriptive sense, and does not amount to a claim that symptomatology should replace existing explanatory frameworks; rather, it is intended to highlight a possible shift in emphasis, whose implications would remain to be clarified empirically and conceptually. Without aiming to advance a new theoretical framework, we wish here to briefly signal a possible opening toward clinical symptomatology as an alternative or complementary candidate for a psychiatric boundary object. Such a proposal would not imply abandoning the medical model (Bolton and Gillett, 2019). The medical model remains explicitly or implicitly embedded in contemporary training and practice (Zachar and Kendler, 2007), and most often framed as biological and cerebral alterations (Kendler, 2012; Huda, 2021). Reorienting the discipline’s focal points and central organizing metaphors toward clinical objects would thus not involve rejecting biological accounts but repositioning them within a broader epistemic framework. From this perspective, it could be conceivable to articulate a form of psychiatry sufficiently stabilized around its own clinical referents to claim a degree of epistemic autonomy, while maintaining substantive connections with the brain sciences.

## Data Availability

The original contributions presented in the study are included in the article/supplementary material, further inquiries can be directed to the corresponding author/s.
